# Vaginal leiomyoma mimicking a Cystocele (report case)

**DOI:** 10.1016/j.ijscr.2022.106955

**Published:** 2022-03-19

**Authors:** Ahmed Touimi Benjelloun, Imane Ziad, Douha Elkaroini, Houssine Boufettal, Sakher Mahdaoui, Naima Samouh

**Affiliations:** Obstetrics and Gynecology Department, University Hospital Center Ibn Rochd, Faculty of Medicine and Pharmacy, Hassan II University of Casablanca, Morocco

**Keywords:** Case report, Ectopic leiomyoma, Vaginal leiomyoma, Cystocele

## Abstract

**Introduction and importance:**

Leiomyoma, known as a disease of the uterus, composed of spindle-shaped smooth muscle fibers and collagenous stroma, is rarely encountered in other sites especially in the vagina.

**Case presentation:**

We report, here, an exceptional case of a vaginal leiomyoma situated in the anterior vaginal wall suggesting in the first place a cystocele. The MRI objectified a rounded formation of the anterior wall of the vagina with regular contours highly suggestive of a vaginal leimyoma. The tumor was surgically removed by the vaginal route. The histopathologic examination confirmed the diagnosis of vaginal leiomyoma.

**Clinical discussion:**

Vaginal leiomyomas are commonly seen in women between the ages of 35 and 50 and are believed to be more common in Caucasian women. Although a rare tumor, vaginal leiomyomas may present with a variety of clinical features and may be mistaken preoperatively for a cystocele, urethrocele, Skene's duct abscess, Gartner's duct cysts, urethral diverticulum, vaginal cysts, cysts Bartholin's gland or a malignant vaginal tumor. The diagnosis is based on careful examination and preoperative imaging (ultrasonography and MRI). Removal of the tumor by vaginal route, wherever possible, with subsequent histopathological examination appears to be the optimum management plan. Although the lesion is benign, local recurrences following incomplete resection and sarcomatous changes have been reported.

**Conclusion:**

Vaginal leiomyoma is a rare benign tumor. The diagnosis is often made only postoperatively after resection of the mass. The tumors may be found in any location within the vagina but are most commonly located on an anterior wall. Imaging can confirm the vaginal origin of the lesion. Surgical excision is the treatment of choice. The diagnosis is based on the histological study of the tumor.

## 1 Introduction

Vaginal tumors are rare and include papilloma, hemangioma, mucosal polyp and rarely leiomyoma. Vaginal leiomyomas are benign mesenchymal tumors that are rare with their ectopic locations, only about 300 cases reported since the first case detected in 1733 by Denys de Leyden [1]. In general, the diagnosis is only made postoperatively, after histological study of the nodule. These tumors most often originate from the anterior vaginal wall causing various clinical presentations or even at the expense of neighboring organs: uterine cervix, bladder, rectum or vulva.

We report a case of a primary leiomyoma of the vagina originating from the anterior wall and presenting with a sensation of an intra-vaginal ball that worsens with prolonged standing and at the end of the day. All our work has been reported in line with the SCARE criteria and guidelines [16].

## 2 Observation

We report the case of a 65-year-old female patient, fifth gesture, fifth pare, mother of five children delivered by vaginal route, who has been menopausal for thirteen years, with no relevant family or personal history, not taking any drug, and who consulted for a vaginal swelling appeared since nine months responsible for a sensation of intra vaginal ball, pelvic heaviness and dyspareunia. This mass progressively increased in size until it became embarrassing. There were no reported episodes of metrorrhagia or functional urinary signs such as retention or urinary incontinence. The general condition was preserved.

**Fig. 1:**
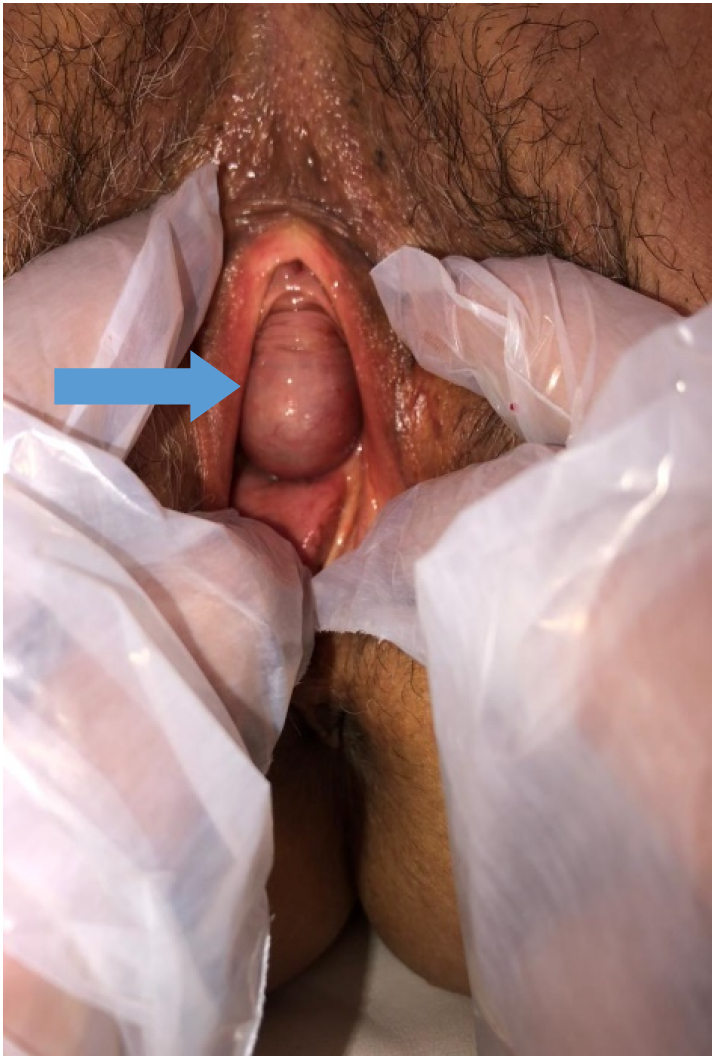
Appearance of vaginal swelling (arrow).

The clinical examination revealed:

• *On inspection of the perineum and vulva*: visualization of a mass in the anterior wall of the vagina 1 cm from the urethral meatus suggestive of a Cystocele.
• *Vaginal touching coupled with abdominal palpation*: palpation of a 3 cm cystic intra vaginal mass, painless, mobile and non reducible. It remains 3 cm away from the anterior cul de sac.
• *On speculum examination*: the cervix appeared normal and no bleeding was observed (Fig. 1).

**Fig. 2:**
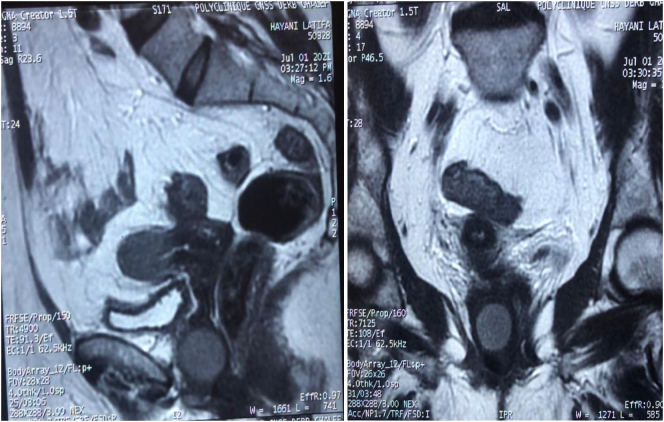
MRI appearance of the intra-vaginal mass.

An endovaginal ultrasound was ordered but did not identify any abnormality. An additional MRI was therefore performed objectifying a rounded formation of the anterior wall of the vagina lateralized to the right measuring 3 cm in diameter with regular contours (Fig. 2).

**Fig. 3:**
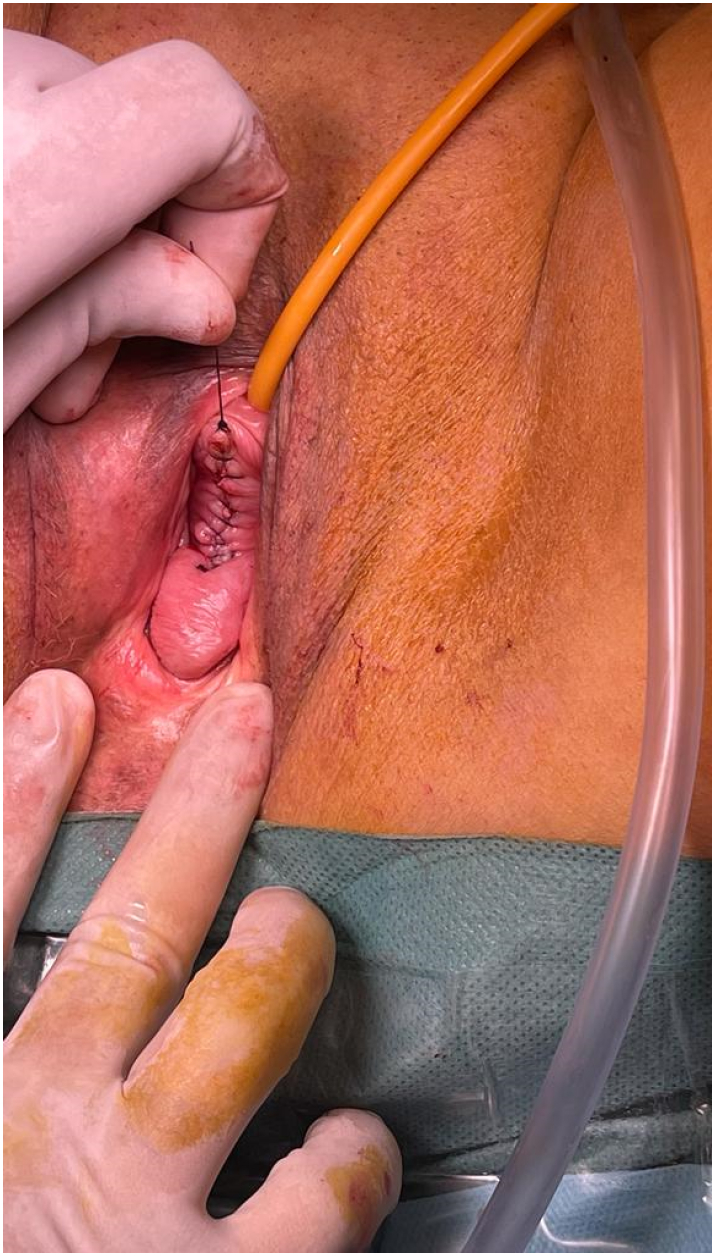
Surgical excision of the lesion.

Given the size of the lesion and its symptomatic nature, a surgical excision was performed by the vaginal route by professor Mahdaoui. A Foley's catheter was introduced in the urethra for protecting the latter. No adhesion was found. We proceeded to the enucleation of the mass and we obliterated the dead space. The surgery took place without incident. The patient was discharged on the second postoperative day. She recovered uneventfully and she was able to resume to her daily activity within the next day. After a two-month follow-up, the vaginal wound healed well and the patient reported no discomfort and was able to resume her sexual activity (Fig. 3).

**Fig. 4:**
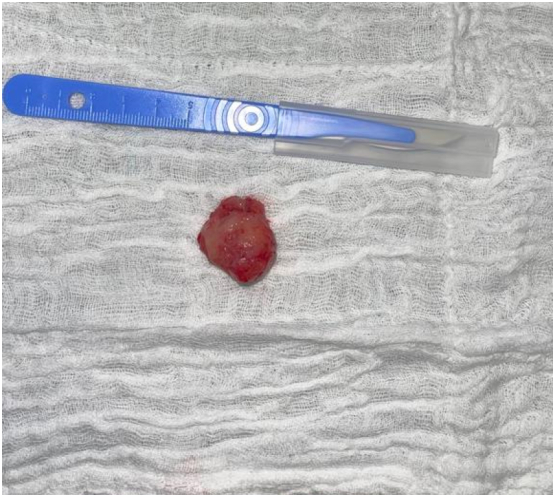
Macroscopic appearance of the lesion.

The tumor was then sent for histopathologic examination. The macroscopic examination describes a solid mass of 2.8 × 2 × 1.8 cm with a whitish fasciculate appearance and the Microscopic examination revealed a well-circumscribed leiomyoma underlying the squamous epithelium, consistent with the diagnosis of vaginal leiomyoma without any signs of malignancy (Fig. 4).

## 3 Discussion

Ectopic leiomyomas are benign mesenchymal tumors that arise from smooth muscle components. They are rare, they can be localized in the ovary, broad ligament, round ligament, and exceptionally in the vulva and vagina [2].

Since the first case described by Denis de Leyde in 1733, around 300 cases of vaginal fibroids have been reported worldwide [1]. Indeed, Bennett and Ehrlich [3] identified only 9 cases out of 50,000 samples for histopathological examination and 1 case out of 15,000 autopsies at Johns Hopkins University. However, Liu [4] argues that the tumor having the characteristics of slow growth and small size, does not produce symptoms at first and regresses spontaneously after menopause, may not manifest in a large number of patients who doesn't become aware of the disease.

Vaginal leiomyomas are commonly seen in women between the ages of 35 and 50 and are believed to be more common in Caucasian women [3], [5].

They usually appear as a single, well-circumscribed mass originating from the medial anterior wall [6], as we have observed in our case, and less frequently, from the posterior and side walls. They are often asymptomatic but depending on the site of onset they can be associated with lower abdominal pain, low back pain, vaginal bleeding, dyspareunia, urinary symptoms as pollakiuria, dysuria or other features of urinary obstruction [7]. The clinical diagnosis of vaginal leiomyoma requires a high index of suspicion as the tumor could easily be mistaken for cystocele, urethrocele, Skene's duct abscess, Gartner's duct cysts, urethral diverticulum, vaginal cysts, cysts Bartholin's gland or a malignant vaginal tumor [8], [9].

The majority of these tumors are around 3 to 4 cm in diameter [10]. The largest vaginal leiomyoma recorded was 20 cm in diameter. In 1933 Pistuddi reported a case of vaginal fibromyoma which, after removal, weighed 1450 g [11]. Usually these tumors are single, benign and slow growing, but sarcomatous transformation has been reported [12].

Imaging, ultrasound and MRI can confirm the vaginal origin of the lesion. On magnetic resonance imaging, they appear as well-demarcated solid masses of low signal intensity on T1 and T2-weighted images, with homogeneous contrast enhancement, while leiomyosarcomas and other vaginal malignancies shows a characteristic high T2 signal intensity with irregular and heterogeneous areas of necrosis or hemorrhage [13]. The use of MRI is particularly useful when fibroids grow rapidly, have poor delimitation on ultrasound, and when there is a high suspicion of malignancy [14]. It is accurate for diagnosing a leiomyoma with a sensitivity of 88 to 93% and a specificity of 66 to 91% [15].

Surgical removal of the mass via the vaginal route remains the therapeutic method of choice, but the abdominoperineal route is necessary for large tumors [5]. The diagnosis is rarely mentioned preoperatively and only the anatomopathological examination makes it possible to retain it. Although the lesion is benign, local recurrences following incomplete resection and sarcomatous changes have been reported [13].

## 4 Conclusion

Vaginal leiomyoma is a benign mesenchymal tumor whose location is rare. This localization is usually asymptomatic, discovered incidentally during a clinical examination or by discomfort or a sensation of intravaginal lump. Vaginal myoma appears as a firm, painless lump, especially on the anterior wall of the vagina. Imaging can confirm the vaginal origin of the lesion. Surgical excision is the treatment of choice. The diagnosis is based on the histological study of the tumor, which can confirm the diagnosis and rule out a malignant component.

## Consent

Written informed consent was obtained from the patient for publication of this case report and accompanying images. A copy of the written consent is available for review by the Editor-in-Chief of this journal on request.

## Provenance and peer review

Not commissioned, externally peer-reviewed.

## Ethical approval

I declare on my honor that the ethical approval has been exempted by my establishment.

## Funding

None.

## Guarantor

Dr. Touimi Benjelloun Ahmed.

## Research registration number

None.

## CRediT authorship contribution statement

Touimi Benjelloun Ahmed: the main doctor conceived the original idea, revised manuscript.
Ziad Imane: followed up, summed up, revised manuscript.
Elkaroini Doha: followed up, wrote manuscript.

All authors contributed to the interpretation of the results, discussed.

## Declaration of competing interest

There are no conflicts of interest.
